# Uric Acid, the End-Product of Purine Metabolism, Mitigates Tau-Related Abnormalities: Comparison with DOT, a Non-Antibiotic Oxytetracycline Derivative

**DOI:** 10.3390/biom15070941

**Published:** 2025-06-28

**Authors:** Bianca Andretto de Mattos, Rodrigo Hernán Tomas-Grau, Thaís Antonia Alves Fernandes, Florencia González-Lizárraga, Aurore Tourville, Ismaila Ciss, Jean-Michel Brunel, Rosana Chehin, Annie Lannuzel, Laurent Ferrié, Rita Raisman-Vozari, Bruno Figadère, Elaine Del Bel, Patrick Pierre Michel

**Affiliations:** 1 Paris Brain Institute-ICM, Inserm, CNRS, APHP, Hôpital de la Pitié Salpêtrière, Sorbonne Université, 75013 Paris, France; biancaandretto@usp.br (B.A.d.M.); rodrigo.tomasgrau@fbqf.unt.edu.ar (R.H.T.-G.); thais.alves.fernandes@usp.br (T.A.A.F.); mflorenciagl@hotmail.com.ar (F.G.-L.); cissismaila87@gmail.com (I.C.); annie.lannuzel@chu-guadeloupe.fr (A.L.); ritaraisman@gmail.com (R.R.-V.); 2 Medical School of Ribeirão Preto, Department of Physiology, University of Sao Paulo, Ribeirão Preto 14040-904, Brazil; eadelbel@usp.br; 3 IMMCA, CONICET-UNT-SIPROSA, Tucumán 4000, Argentina; rosana.chehin@fbqf.unt.edu.ar; 4 BioCIS, CNRS, Université Paris-Saclay, 91400 Orsay, France; laurent.ferrie@universite-paris-saclay.fr (L.F.); bruno.figadere@universite-paris-saclay.fr (B.F.); 5 Inserm, Membranes et Cibles Thérapeutiques, Service de Santé des Armées, Aix Marseille Université, 13385 Marseille, France; bruneljm@yahoo.fr; 6 Department of Neurology, CIC1424, University Hospital of Guadeloupe, Antilles University, French West Indies, 97159 Pointe-à-Pitre, France; 7 Dentistry School, Basic and Oral Biology, University of Sao Paulo, Ribeirão Preto 14040-904, Brazil

**Keywords:** aggregation, Alzheimer disease, excitotoxicity, hemin, iron, non-antibiotic tetracyclines, oxidative stress, phospho-tau, tauopathies

## Abstract

We aimed to simulate tau abnormalities—specifically hyperphosphorylation and aggregation—that are hallmarks of tauopathies, including Alzheimer’s disease, to evaluate tau-targeting therapies. To model pathological p-tau accumulation at early disease stages, we exposed mouse cortical cultures to redox-active iron from hemin (Hm), a breakdown product of hemoglobin, or challenged them with the excitatory neurotransmitter glutamate. Using the AT8 phospho-specific antibody, we demonstrate that a subtoxic concentration of Hm (3 µM) promotes pathological p-tau accumulation in a subpopulation of cultured cortical neurons and their proximal neurites. Uric acid (UA; 0.1–200 µM), the metabolic end-product of purines in humans, prevented p-tau build-up. Neither xanthine, the immediate precursor of UA, nor allantoin, its oxidized product, reproduced this effect. Live cell imaging studies revealed that UA operates by repressing iron-driven lipid peroxidation. DOT (3 µM), a brain-permeant tetracycline (TC) without antibiotic activity, mimicked UA’s anti-tau and antioxidant effects. Interestingly, both UA and DOT remained effective in preventing p-tau accumulation induced by glutamate (10 µM). To simulate tau aggregation at more advanced disease stages, we conducted a Thioflavin-T aggregation assay. Our findings revealed that UA and DOT prevented tau aggregation seeded by heparin. However, only DOT remained effective when heparin-assembled tau fibrils were used as the seeding material. In summary, our results indicate that UA-elevating agents may hold therapeutic utility for tauopathies. The non-purine compound DOT could serve as an effective alternative to UA-related therapies.

## 1. Introduction

Tauopathies belong to a heterogeneous class of age-related neurodegenerative disorders primarily characterized by the accumulation of insoluble proteinaceous aggregates made of abnormally phosphorylated tau [1,2]. Aggregates of misfolded tau protein forming neurofibrillary tangles (NFTs) are believed to be implicated in neuronal dysfunction and death, leading to a range of symptoms including cognitive decline, behavioral changes, dementia, and motor deficits [3,4]. It has been hypothesized that neurodegeneration in tauopathies results from a loss of tau’s ability to stabilize microtubule assemblies, but it is conceivable that other cellular functions are also perturbed during tau pathological processes [5].

Tauopathies can be classified as primary or secondary depending on whether the tau pathology appears as the main driver of neurodegeneration. Primary tauopathies include corticobasal degeneration, progressive supranuclear palsy, frontotemporal lobar degeneration with tau pathology, Pick’s disease, and argyrophilic grain disease [3,6,7]. Alzheimer’s disease (AD), the most well-studied tauopathy, is classified as a secondary tauopathy as tau NFT formation follows the appearance of amyloid-β protein deposits. Secondary tauopathies may also have an environmental cause, such as trauma in chronic traumatic encephalopathy [7,8] or toxins in atypical forms of degenerative Parkinsonism [9,10].

In AD, it is believed that tau aggregation and the formation of NFTs are preceded by a pre-tangle stage during which non-fibrillary hyperphosphorylated tau accumulates in the soma and dendrites of vulnerable neurons [11,12,13]. This suggests that tau hyperphosphorylation may be an initiating step for tau aggregation into NFTs [14,15,16].

In this study, we aimed to simulate tau abnormalities occurring in the early and advanced stages of AD and other tauopathies to identify new tau-directed therapies. To model the early stages of pathological tau build-up, we established mouse primary cortical cultures and exposed them to redox-active iron from hemin (Hm), a breakdown product of hemoglobin (Hb) [17,18], or to low excitotoxic levels of the neurotransmitter glutamate [19]. The selection of these two cellular stressors was motivated by the fact that iron dyshomeostasis [20,21] and neuronal hyperexcitability [22,23] are believed to actively contribute to the pathophysiology of tauopathies. For cellular immunodetection of pathological tau, we used the AT8 phospho-specific antibody, which enables tracking of the progression of a pathological tau burden in the human brain by specifically detecting phospho-tau (p-tau) Ser202/Thr205 residues [16,24,25].

Our findings revealed that p-tau accumulates in certain subsets of neuronal cell bodies and their proximal neurites, enabling us to test molecules that could potentially prevent these pathological events. Specifically, we evaluated the effects of uric acid (UA), which is the metabolic end-product of purines. Investigating UA in this context is of interest because lower circulating levels of this purine may represent a risk factor for AD dementia and other tauopathies [26,27]. We compared the effect of UA to that of a newly designed non-antibiotic TC having potent neuroprotective/antioxidant effects in experimental paradigms that model Parkinson’s disease (PD) neurodegeneration [28].

To reproduce tau abnormalities at a more advanced disease stage, we monitored the aggregation of 2N4R tau monomers in a Thioflavin-T fluorescent assay. Specifically, test compounds were evaluated when tau aggregation was seeded by heparin or heparin-assembled tau fibrils [29,30].

## 2. Materials and Methods

### 2.1. Use of Animals

The mice used were housed, handled, and cared for in strict accordance with the European Union Council Directives (2010/63/EU). The Committee on the Ethics of Animal Experiments Charles Darwin no. 5 approved experimental protocols under the authorization number Ce5/2024/001.

### 2.2. Cortical Neuronal Cultures

We established primary cultures of cortical neurons from Swiss mouse embryos at day 13.5 of gestation (Janvier LABS; Le Genest St Isle, France). Briefly, brain cortices were dissected out and then incubated for 20 min at 37 °C in an EDTA (2 mM)–trypsin (0.05%) solution for brain tissue digestion before trituration. Then, trypsin was neutralized with Dulbecco’s Modified Eagle’s Medium (DMEM; Thermo Fisher Scientific, Courtaboeuf, France) containing 10% fetal calf serum (FCS; Biowest LLC, Les Ulis, France), and mechanical trituration performed in Leibovitz L15 culture medium (Thermo Fisher Scientific; Courtaboeuf, France) following protocols previously described in detail for midbrain cultures [28,31]. Dissociated cells in suspension were seeded at a density of 40–60 × 10^3^ cells/cm^2^ onto Nunc 48-well multiwell plates (Roskilde, Denmark) or 8-well glass bottom µ-slides (#80807; Ibidi, Gräfelfing, Germany) pre-coated with 1 mg/mL polyethylenimine (PEI; P3143; Sigma Aldrich, L’Isle-d’Abeau Chesnes, France ) dissolved in a pH = 8.3 borate buffer as described before [32,33].

Cell culture was initiated in Neurobasal-A medium (Nb; #10888022; Gibco, Saint Aubin, France) supplemented with a B27 cocktail without antioxidants (#10889038; Gibco), an N2 mix (#17502048; Gibco), 100 IU/mL of penicillin/streptomycin (#15140122; Gibco), and 1% FCS. This medium is referred to as modified Nb (mNb). After plating, the cultures were treated daily with 1.5 µM of the antimitotic agent cytosine arabinoside (Ara-C; Sigma Aldrich) to reach a cumulative concentration of 4.5 µM at day in vitro (div)3. This treatment regimen, which is not toxic for neuronal cells, allows us to eliminate >95% of glial cells from the cultures [34,35]. At the end of div3, the plating medium was completely substituted by mNb medium lacking FCS, which had been previously conditioned in pure astrocyte cultures [31,36]. Cortical cultures were then maintained in astrocyte-conditioned medium (ACM) until termination of the cultures.

**Figure 1:**
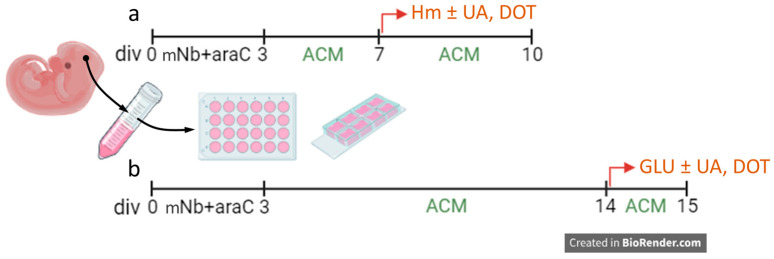
Experimental conditions implemented to promote tau abnormalities in mouse cortical neurons in culture. (**a**) Cortical cultures treated with hemin (Hm) on div7, 8, and 9, and then processed at div10 for analysis. (**b**) Div14 cortical cultures challenged with 10 µM of glutamate (GLU) for 24 h and then processed for analysis. In each experimental paradigm, we evaluated the capacity of UA, DOT, and the other test compounds to prevent p-tau accumulation. ACM: astrocyte-conditioned medium. mNb: modified neurobasal medium. Created with Biorender.com.

To model tau-related neurodegenerative changes, we treated the cultures with hemin (Hm), a breakdown product of Hb [17,18], which contains redox-active iron, or with the excitatory neurotransmitter glutamate [19]. When exposing cortical cultures to Hm, treatments were carried out on div7, 8, and 9, and the consequences of such treatments were evaluated at div10. When tau neurodegenerative changes were provoked by a challenge with glutamate, we used div14 cortical cultures and a concentration of the excitatory neurotransmitter of 10 µM, causing the partial neurodegeneration of cortical neurons. In that case, cultures were taken for analysis at div15. The two protocols used to evaluate the anti-tau effects of test treatments are described in Figure 1.

### 2.3. The Non-Antibiotic TC Compound, DOT

#### 2.3.1. Synthesis of DOT

The non-antibiotic TC compound, 4-dedimethylamino-12a-deoxy oxytetracycline (DOT), was synthesized in-house using a protocol previously described [28]. The synthesis strategy is a modification of that initially developed by Golub and colleagues [37] to generate non-antibiotic TC derivatives. Specifically, we removed the dimethylamino substituent at position 4 to eliminate the antimicrobial activity, along with the hydroxy group at position 12a on ring A of oxytetracycline [28]. Stock solutions of DOT dissolved at 50 mM in DMSO were kept at −20 °C for less than 6 months. Intermediate dilutions used for cell culture treatments were made in distilled water and stored for 7 days at 4 °C, protected from light.

#### 2.3.2. Capacity of DOT to Penetrate the Brain

The efficacy of DOT in penetrating the brain was assessed using adult Swiss mice receiving a single subcutaneous injection of 30 mg/kg of DOT diluted in saline with 5% DMSO and 5% Tween 80. This formulation allowed administration in a volume of 4 mL/kg. After being sacrificed 30 min, 1 h, 8 h, and 24 h after treatment, brain and serum samples (n = 3/time point) were collected and mixed with acetonitrile for compound extraction. After vortexing and sonication, proteins and solid residues were removed by centrifugation (15,000× *g*, 5 min), and supernatants were analyzed using a UHPLC system coupled with a triple quadrupole mass spectrometer LCMS-8030 (Shimadzu Corporation, Kyoto, Japan). The brain-to-plasma ratio calculated from the area under the concentration–time curves in the brain and plasma was 0.6 ± 0.2 (n = 3), which demonstrates the good brain penetration of this compound.

### 2.4. Uric Acid and Related Purine Derivatives

UA (#U2625), its immediate precursor xanthine (XANT; #X7375), its oxidation product allantoin (ALTN; #93791), and its synthetic analog 1, 7-dimethyluric acid (DMUA; #40407) were obtained from Sigma Aldrich (L’Isle-d’Abeau Chesnes, France). Stock solutions of UA, XANT, DMUA, and ALTN were all made at 10 mM. UA was first solubilized in 1N NaOH before bringing the pH of the solution to ~7 using 1N HCl. XANT and DMUA were diluted using the same procedure, whereas ALTN was diluted in sterile distilled water.

### 2.5. Other Pharmacological Reagents

Hemin (Hm; #51280) was diluted in DMSO to obtain a 20 mM stock solution and intermediate dilutions were made fresh in distilled water just before use. Stock solutions of inhibitors of lipid peroxidation/ferroptosis Trolox-C (TROL; #238813) and Liproxstatin-1 (LIP; #6113) were made at 50 mM in pure ethanol and DMSO, respectively. The iron chelator desferoxamine (DESF; #D9533) and vitamin C (VitC; #255564) were diluted at 10 mM in distilled water. The iron-chelating glycoprotein (APO, #T1428) was dissolved in distilled water at a concentration of 10 mg/mL.

### 2.6. Immunocytochemical Procedures

After the termination of test treatments, cortical cultures were fixed for 12 min in Dulbecco’s phosphate-buffered saline (PBS) containing 4% formaldehyde (#252549; Sigma Aldrich), washed with PBS, and then incubated for 18 h with a mouse p-tau monoclonal antibody (AT8) (MN1020, Thermofisher Scientific, Courtaboeuf, France; 1:500 in 0.2% Triton X-100/PBS), which was revealed with an anti-mouse IgG (H + L) conjugated to Alexa Fluor 488 (#A11001 Thermofisher Scientific). Then, cortical cultures were incubated for 18 h with a chicken anti-microtubule-associated protein-2 (MAP-2) antibody (ab5392, Abcam, Cambridge, UK; 1:1000 in PBS), which was revealed with an anti-chicken IgG (H + L) conjugated to Alexa Fluor 555 (#A32932, Thermofisher Scientific).

### 2.7. Cell Counting Procedures

For cell-counting operations, we used a Nikon Eclipse Ti-U fluorescence inverted microscope (Nikon France, Champigny sur Marne, France) equipped with an ORCA-Flash digital camera (Hamamatsu Photonics, Massy, France) and the NIS-Elements software Version 5.41 (Nikon). The number of p-tau^+^ cell bodies and MAP-2^+^ neurons was estimated by taking microphotographs with a 40× objective of 5–10 visual fields that were randomly selected for each treatment condition. Cell-counting operations were performed with the open-source FIJI software (version 2.1.0/1.54p) [38]. No blinding procedure was undertaken.

### 2.8. Confocal Imaging

Confocal imaging was performed using a Nikon A1R HD25 microscope (Nikon, Amstelveen, The Netherlands) equipped with the NIS-Elements software (Version 5.41; Nikon). Images from cultures grown on glass-bottom Ibidi μ-slides were acquired every 0.38 μm in the Z direction using a 40× water immersion objective (NA 1.3; WD 0.2 mm). All acquisitions were performed under the resonant scanner mode. Image reconstructions were performed with the FIJI software.

### 2.9. Assessment of ROS Emission and Changes in Mitochondrial Membrane Potential

To assess changes in reactive oxygen species (ROS) and mitochondrial membrane potential (ΔΨm), the culture medium was removed and immediately replaced by warm PBS–glucose (5 mM). Then, the cultures were exposed to tetramethylrhodamine methyl ester, perchlorate (TMRM; 50 nM; ab228569; Abcam, Cambridge, UK), and dihydrorhodamine 123 (DHR-123; 25 µM; D23806; Thermo Fisher Scientific) 10 min later. After 35 min, the cultures were washed extensively (3×) with PBS–glucose to remove fluorescent probes in excess before carrying out live cell imaging [35,39,40]. In some experiments, ROS production was induced with the prooxidant compound H_2_O_2_ (50 µM; #8070.4; CARL ROTH, Karlsruhe, Germany) and mitochondrial depolarization with the protonophore carbonyl cyanide 4-(trifluoromethoxy)phenylhydrazone (FCCP; 0.5 µM) provided in the TMRM assay kit. H_2_O_2_ and FCCP were added 4 h and 10 min before adding fluorogenic probes, respectively. Note that FCCP, but not H_2_O_2,_ was present throughout the incubation in PBS–glucose.

For each culture condition, fluorescent images from at least five randomly chosen fields were acquired with a 40× fluorescence objective using a Nikon Eclipse Ti-U fluorescence inverted microscope equipped with an ORCA Flash digital camera. The excitation and emission wavelengths for DHR-123 were 490 nm and 525 nm, respectively, whereas the corresponding wavelengths for TMRM were 548 nm and 575 nm, respectively. The open-source FIJI software was used for quantifying fluorescent signal intensities [38] over the surface area of each individual cell body morphologically identifiable by phase contrast (Phaco) optics [28]. The results were expressed in changes in fluorescence intensity relative to non-treated cultures.

### 2.10. Tau Aggregation Assay

For in vitro aggregation assays, we utilized recombinantly expressed full-length human tau (2N4R) obtained from BrinDx (www.brindx.com (accessed on 22 October 2024); #BRX-2002). Aggregation assays were based on protocols reported previously [29,41]. Briefly, samples containing tau monomers (tau_m_; 22 µM) were resuspended in PBS and mixed with the polyanionic cofactor heparin (0.2 mg/mL) along with ThT (10 µM), a fluorescent reporter molecule used to monitor the formation of β-sheet-rich fibril structures [42]. Heparin-assembled tau fibrils (tau_f_) (2.2 µM monomer equivalent) were also used as seeds instead of heparin.

Fibrillar aggregates of tau were generated by incubating test samples in an orbital shaker (Thermomixer Comfort; Eppendorf, Montesson, France) at a speed of 600 rpm for 72 h. After this incubation, end-stage products were measured by fluorescence emission with a Fluoromax-4 spectrofluorometer, setting the excitation wavelength at 450 nm. Prior to these measurements, each test molecule was subjected to standard biophysical assessments—specifically absorbance and fluorescence analyses—to confirm that it did not emit fluorescence within the spectral range of ThT. This precaution ensured that any detected ThT signal variations could be confidently attributed to conformational changes in tau and not to spectral interference from the test compound.

### 2.11. Statistical Analysis

Data are presented as the mean ± SEM. Statistical outliers were removed using the ROUT method (Q = 5%) [43]. The normality or near-normality of the datasets was checked by Shapiro–Wilk testing or QQ plot visualization, respectively [44]. When normality was assumed, we conducted a one-way ANOVA followed by a Tukey’s test for all pairwise multiple comparisons or a Dunnett’s test for multiple comparisons against a single control group. In cases where normality could not be assumed, data were analyzed with a Kruskal–Wallis ANOVA on ranks, followed by Dunn’s multiple comparison test. A *p*-value of <0.05 was considered statistically significant. Statistical evaluation of the data is presented in the figure legend.

## 3. Results

### 3.1. Induction of P-Tau Abnormalities by Hemin

We aimed to establish an experimental cell culture model reproducing the accumulation of hyperphosphorylated tau, which is characteristic of tauopathies [14,15]. To achieve this, we utilized cortical cultures that were chronically exposed to redox-active iron from Hm. Specifically, cortical cultures maintained in ACM were treated repeatedly at div7, 8, and 9 with concentrations of Hm ranging from 3 to 30 µM with no medium change between treatments, allowing for final cumulative concentrations of 9 to 90 µM by div10, when the cultures were processed for analyses (Figure 1).

**Figure 2:**
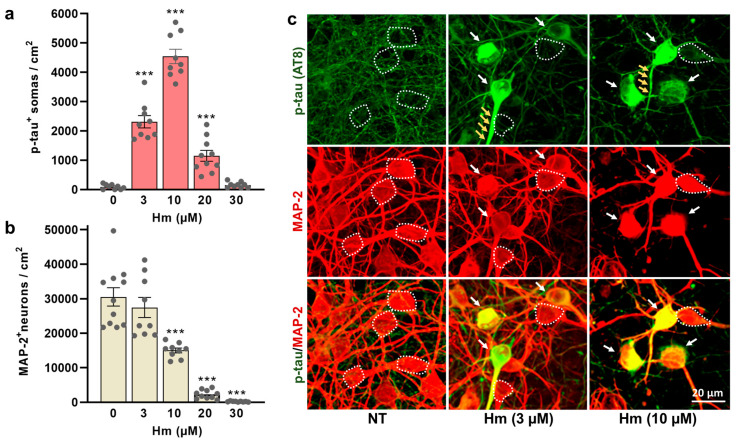
Induction of p-tau-related neurodegenerative changes by Hm. (**a**,**b**) Number of p-tau^+^ somas (AT8 immunosignal) (**a**) and MAP-2^+^ neurons (**b**) in div10 cortical cultures treated repeatedly at div7, 8, and 9 with 3–30 µM of Hm. (**a**,**b**) Data expressed in numbers of p-tau^+^ somas/cm^2^ or neuronal cells/cm^2^ in div10 cultures are presented as the mean ± SEM. One-way ANOVA followed by Tukey’s test, *** *p* < 0.001 vs. NT. (**c**) Single-color and merged images from div10 cortical cultures repeatedly exposed to Hm (3 and 10 µM) and then immunolabelled for p-tau (green) and MAP-2 (red). White arrows indicate cortical neurons accumulating p-tau in their somas. Small yellow arrows (upper panel) point to neuritic extensions where the p-tau immunosignal is strongly increased. The white dotted line shows the boundaries of neuronal cell bodies with a near absence of p-tau immunostaining. NT: non-treated.

The induction of p-tau by Hm is described in Figure 2a–c. Precisely, we show that the overall number of neuronal somas accumulating tau phosphorylated at the AT8 epitope is relatively low (though not null) in control cortical cultures (Figure 2a). A faint and punctuated AT8 immunosignal was also detectable in the neurite network of the control cultures (Figure 2c), which is indicative of basal levels of AT8 tau phosphorylation in neurites under these conditions. There was, however, a sharp increase in the number of cell bodies accumulating hyperphosphorylated tau when the cultures were exposed to 3 to 10 µM of Hm for three consecutive days. We found that the pathological accumulation of p-tau in neuronal somas was significant at 3 µM and reached its peak at 10 µM of Hm, before decreasing at higher concentrations (Figure 2a). Neuronal death was not significant at 3 µM of Hm (Figure 2b). However, at concentrations of 10 µM Hm and higher, we observed a gradual decline in neuronal survival, reaching a peak at 30 µM. Note that at 3 and 10 µM of Hm, a strong AT8 immunosignal was also observed in proximal neurites originating from cell bodies, accumulating p-tau. The impact of repeated treatment regimens with Hm (3 and 10 µM) on p-tau accumulation and neuronal survival is illustrated by microphotographs from div10 cortical cultures (Figure 2c). In subsequent experiments, we applied a treatment regimen using Hm at a concentration of 3 µM to simulate p-tau accumulation without significantly affecting neuronal survival.

### 3.2. Hm-Induced P-Tau Abnormalities Are Curtailed by the Purine Metabolic End-Product UA

**Figure 3:**
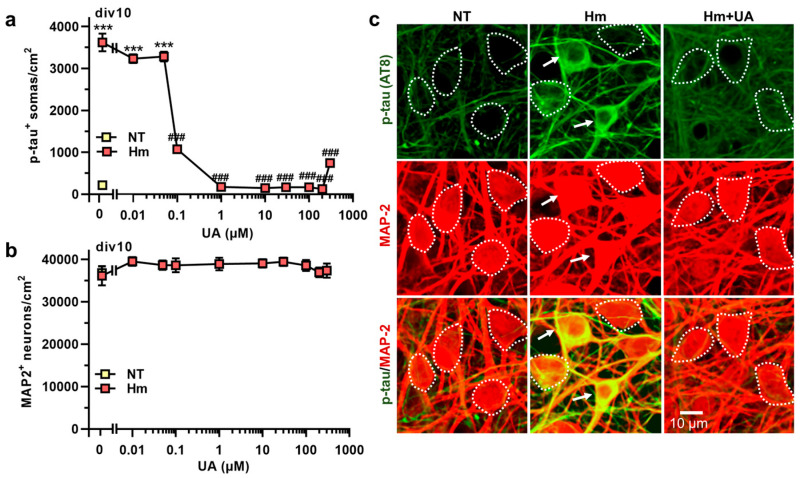
p-tau build-up induced by Hm is curtailed by uric acid. (**a**) Number of p-tau^+^ somas (AT8 immunosignal) in div10 cortical cultures treated repeatedly at div7, 8, and 9 with 3 µM of Hm, together or not with 0.01–300 µM of UA. (**b**) Survival of MAP-2^+^ neurons in div10 cortical cultures exposed to the same treatments as in (**a**). (**a**,**b**) Data expressed in numbers of p-tau^+^ somas/cm^2^ or MAP-2^+^ neurons/cm^2^ in div10 cultures are presented as the mean ± SEM (n = 6–9). One-way ANOVA followed by Tukey’s test: *** *p* < 0.001 vs. NT. ^###^ *p* < 0.001 vs. Hm. (**c**) Representative microphotographs illustrating the inhibitory effects of 30 µM UA on the accumulation of p-tau in the soma of MAP-2^+^ neurons treated with 3 µM Hm. Note that neuronal survival is not impacted by any of the test treatments. White arrows point to cortical neurons accumulating p-tau in their somas. The white dotted line shows the boundaries of neuronal cell bodies with a near absence of p-tau immunostaining. NT: non-treated.

We initially aimed to investigate whether the end-product of purine metabolism in humans, UA, could counteract p-tau accumulation after Hm exposure. To achieve this, cortical cultures that were repeatedly exposed to 3 µM Hm were concomitantly treated with varying concentrations of UA (0.01–300 µM). As shown in Figure 3a, a concentration of 0.1 µM UA reduced the number of cortical neuronal cell bodies accumulating p-tau by about 70%. UA demonstrated optimal anti-tau effects between 1 and 200 µM, reducing the number of p-tau^+^ somas by over 95% within this range of concentrations. At 300 µM, UA appeared to lose some of its efficacy (Figure 3a). Importantly, we did not observe any neuronal loss, regardless of the treatment applied to cultures (Figure 3b). Figure 3c illustrates the inhibitory effects of 30 µM UA against Hm-induced p-tau accumulation.

### 3.3. UA Exerts Anti-Tau Effects by Curtailing Hm-Mediated Oxidative Insults

**Figure 4:**
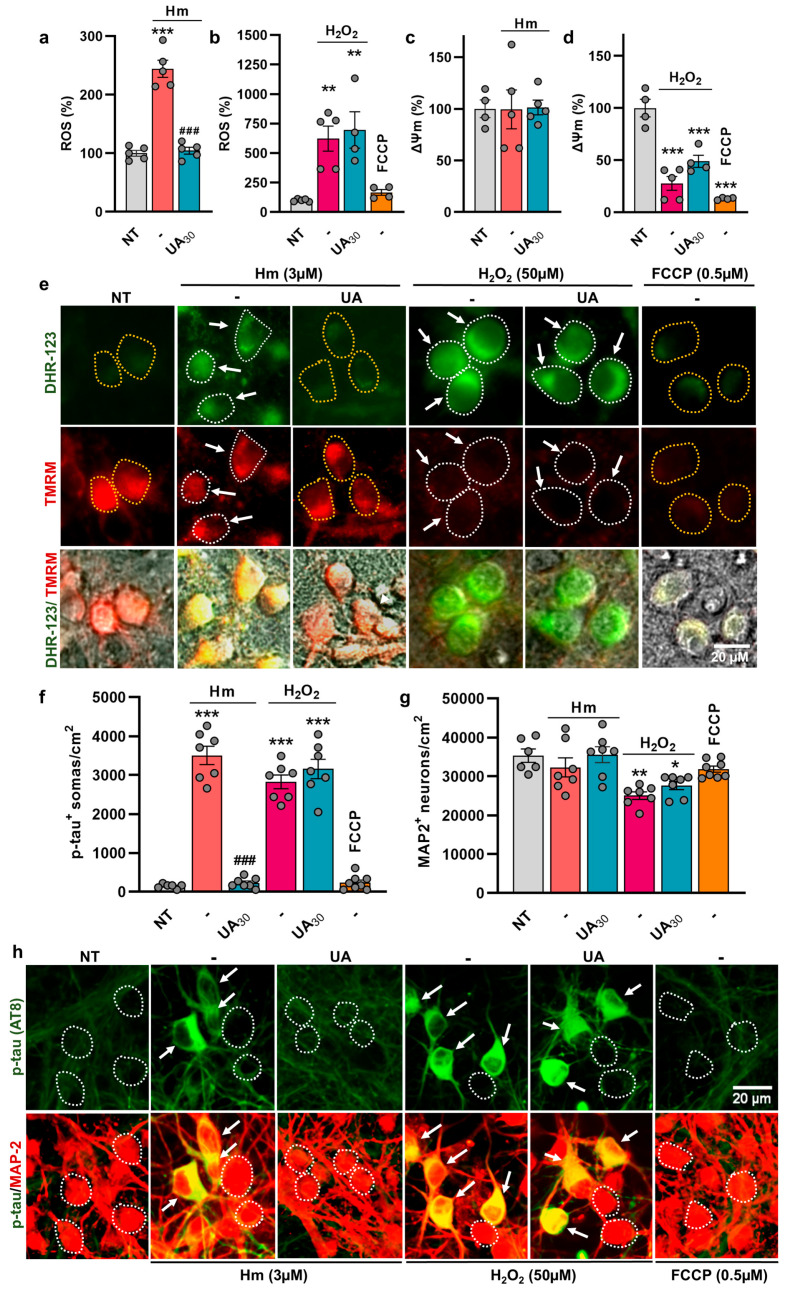
UA represses Hm-induced p-tau build-up by curtailing ROS-mediated insults. (**a**) ROS emission in div10 cortical cultures treated repeatedly with 3 µM Hm in the presence or not of 30 µM UA. (**b**) Comparison with cultures acutely exposed for 4 h to H_2_O_2_ (50 µM) in the presence or the absence of 30 µM UA. The impact of an acute challenge with 0.5 µM of the protonophore FCCP is also illustrated. (**c**) Estimation of ΔΨm in div10 cortical cultures treated as in (**a**). (**d**) Estimation of ΔΨm in div10 cortical cultures treated as in (**b**). (**a**–**d**) Data expressed in % of NT-cultures are presented as the mean ± SEM. One-way ANOVA followed by Tukey’s test: ** *p* < 0.01 and *** *p* < 0.001 vs. NT; ^###^ *p* < 0.001 vs. Hm. (**e**) Representative images showing DHR-123 (upper panel) and TMRM (mid panel) fluorescent signals in div10 cortical cultures receiving the same treatments as in (**a**–**d**). The lower panel represents Phaco images merged with the DHR-123 and TMRM fluorescent signals. Neuronal cell bodies exhibiting increased ROS levels are delineated by a white dotted line and pointed out by a white arrow. Neuronal cell bodies exhibiting basal ROS levels are delineated by a yellow dotted line. (**f**) Estimation of p-tau build-up in div10 cortical cultures receiving the same treatments as in (**a**–**d**). (**g**) Estimation of neuronal survival in div10 cortical cultures receiving the same treatments as in (**a**–**d**). (**f**,**g**) Data expressed in numbers of p-tau^+^ somas/cm^2^ or MAP-2^+^ neurons /cm^2^ in div10 cultures are presented as the mean ± SEM. One-way ANOVA followed by Tukey’s test: * *p* < 0.05, ** *p* < 0.01, and *** *p* < 0.001 vs. NT; ^###^ *p* < 0.01 vs. Hm. (**h**) Representative photomicrographs of div10 cortical cultures showing that UA prevents p-tau build-up induced by Hm but not H_2_O_2_. White arrows point to cortical neurons accumulating p-tau in their somas. The white dotted line shows the boundaries of neuronal cell bodies with a near absence of p-tau immunostaining.

Because UA is known to operate as a potent antioxidant [45,46], we wished to determine whether the anti-tau effects of UA could result from the inhibition of ROS emission. To this aim, we used the ROS-sensitive dye DHR-123 for monitoring intracellular ROS production in Hm (3 µM)-treated cultures exposed or not to UA (30 µM), compared to NT cultures (Figure 4a). Precisely, we show that a treatment with 3 µM Hm is associated with an increase in ROS production and that a concomitant treatment with 30 µM UA totally prevents this effect. We also demonstrate that ROS emission is stimulated even more vigorously when div10 cortical cultures are acutely challenged with 50 µM H_2_O_2_ for 4 hrs. In that case, however, UA (30 µM) could not reduce ROS production (Figure 4b).

Concomitant to ROS quantification with DHR-123, we monitored changes in ΔΨm that could occur in ROS-emitting neurons [35,40]. Using the mitoprobe TMRM, we show that the ROS signal induced by 3 µM Hm is not associated with significant changes in ΔΨm (Figure 4c). ΔΨm was similarly preserved in UA-treated cultures. There was a drop in ΔΨm, however, when cortical cultures were challenged with H_2_O_2,_ regardless of the presence of UA (Figure 4d). An acute challenge with the protonophore FCCP (0.5 µM), which operates as an uncoupler of mitochondrial oxidative phosphorylation, led to an expected drop in ΔΨm (Figure 4d) but failed to stimulate ROS production (Figure 4b). Figure 4e provides representative illustrations showing the DHR-123 (upper panel) and TMRM (mid panel) fluorescent signals in div10 cortical cultures treated repeatedly with 3 µM Hm or acutely with H_2_O_2_ in the presence or not of 30 µM UA. The acute challenge with FCCP is also illustrated in comparison. The lower panel represents Phaco images merged with the DHR-123 and TMRM fluorescent signals.

As expected, treatment with 30 µM UA prevented p-tau build-up induced by 3 µM of Hm (Figure 4f). However, UA failed to curtail p-tau accumulation induced by acute exposure to H_2_O_2_. The treatment with FCCP, causing a reduction in ΔΨm and no increase in ROS production, failed to promote p-tau accumulation (Figure 4f). This indicates that the transient dissipation of ΔΨm cannot lead per se to p-tau accumulation.

Cell counting of MAP-2^+^ cell bodies confirmed that neuronal survival was not affected by Hm (3 µM) exposure, whether UA (30 µM) was present or not in these cultures (Figure 4g). On the contrary, a 4 h challenge with 50 µM H_2_O_2_ led to a 25% decrease in neuronal survival that was not compensated by UA. Finally, the acute challenge with 0.5 µM FCCP did not result in significant neuronal loss in the present experimental time frame. Photomicrographs illustrate the impact of various test treatments on p-tau accumulation in div10 cortical cultures (Figure 4h).

### 3.4. The Suppression of P-Tau Build-Up by UA Is Reproduced by Compounds Inhibiting Iron-Mediated Lipid Peroxidation

**Figure 5:**
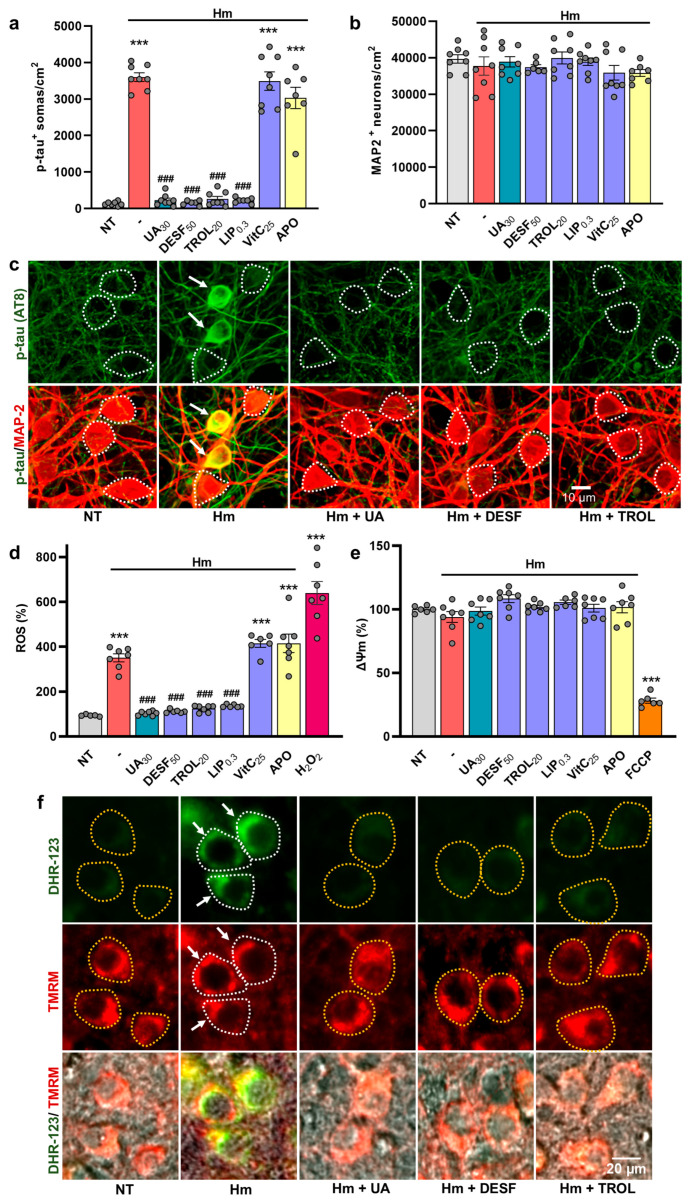
Prevention of Hm-induced p-tau build-up by UA is reproduced by compounds inhibiting iron-mediated lipid peroxidation. (**a**) Estimation of p-tau accumulation (AT8) in div10 cortical cultures treated repeatedly at div7, 8, and 9 with 3 µM Hm in the presence or not of UA (30 µM), the iron chelator DESF (50 µM), the inhibitors of lipid peroxidation and ferroptosis TROL (20 µM) and LIP (0.3 µM), the water-soluble vitamin VitC (25 µM), or the glycoprotein APO (100 µg/mL). (**b**) Estimation of neuronal survival in div10 cortical cultures undergoing the same treatments as in (**a**). (**a**,**b**) Data expressed in numbers of p-tau^+^ somas/cm^2^ or MAP-2^+^ neurons/cm^2^ in div10 cultures are presented as the mean ± SEM. One-way ANOVA followed by post hoc Tukey’s test: *** *p* < 0.001 vs. NT, ^###^ *p* < 0.001 vs. Hm. (**c**) Upper panel: Representative photomicrographs showing the impact of UA (30 µM), DESF (50 µM), and TROL (20 µM) on Hm (3 µM)-induced p-tau accumulation in div10 cortical cultures. Lower panel: Same cell culture field in which the p-tau immunosignal is combined with MAP-2 immunolabeling. White arrows point to cortical neurons accumulating p-tau in their somas. The white dotted line shows the boundaries of neuronal cell bodies with a near absence of p-tau immunostaining. (**d**) Estimation of ROS emission in div10 cortical cultures treated repeatedly at div7, 8, and 9 with 3 µM Hm in the presence or not of UA (30 µM) and the other test treatments described in (**a**). The oxidizer H_2_O_2_ (50 µM), acutely applied to the cultures for 4 h, is used as a positive control for ROS emission. (**e**) Estimation of ΔΨm in div10 cortical cultures treated repeatedly at div7, 8, and 9 with 3 µM Hm in the presence or not of UA (30 µM) or the other test treatments described in (**a**). The protonophore FCCP (0.5 µM) applied acutely to the cultures is used as a reference compound to induce ΔΨm dissipation. (**d**,**e**) Data expressed in % of NT-cultures are presented as the mean ± SEM. One-way ANOVA followed by post hoc Tukey’s test: *** *p* < 0.001 vs. NT, ^###^ *p* < 0.001 vs. Hm. (**f**) Representative images showing DHR-123 (upper panel) and TMRM (mid panel) fluorescent signals in div10 cortical cultures treated with 3 µM Hm in the presence or not of UA (30 µM), DESF (50 µM), or TROL (20 µM). The lower panel represents Phaco images merged with DHR-123 and TMRM fluorescent signals. Neuronal cell bodies exhibiting increased ROS levels are delineated by a white dotted line and pointed out by a white arrow. Neuronal cell bodies exhibiting basal ROS levels are delineated by a yellow dotted line.

To better understand the nature of the inhibitory effects of UA on p-tau accumulation, we performed a series of experiments comparing its suppressive action to that of other treatments susceptible of mimicking the effects of the purine compound. These treatments comprise the iron chelator DESF (50 µM), the inhibitors of lipid peroxidation and ferroptosis TROL (20 µM) and LIP (0.3 µM), the water-soluble vitamin VitC (25 µM), and APO (100 µg/mL) an iron-carrying glycoprotein reported to be protective against iron-mediated neurodegeneration [35,47]. We showed that DESF, TROL, and LIP mimicked the anti-tau effects of UA in cortical cultures treated repeatedly with 3 µM Hm. The efficacy of DES, TROL, and LIP was comparable to that of 30 µM UA (Figure 5a). VitC and APO, however, did not exert anti-tau effects in this setting. No neuronal cell loss was observed regardless of the treatments applied (Figure 5b). Photomicrographs from Figure 5c illustrate the impact of various test treatments on p-tau accumulation in div10 cortical cultures exposed to 3 µM Hm.

Then, we monitored ROS production and changes in ΔΨm in cultures receiving the same treatments as before. As expected, intracellular oxidative stress was significantly elevated in cortical cultures receiving a treatment regimen with 3 µM Hm. Conversely, ROS returned to basal levels when DESF, TROL, and LIP were added to Hm-treated cultures instead of UA (Figure 5d). However, APO and VitC were ineffective in reducing ROS production induced by Hm. ΔΨm was preserved whatever the test treatments applied to the cultures (Figure 5e). As expected, the oxidizer H_2_O_2_ (50 µM) caused a large increase in ROS emission and the protonophore FCCP (0.5 µM) a decrease in ΔΨ (Figure 5d,e).

Figure 5f provides representative illustrations showing DHR-123 (upper panel) and TMRM (mid panel) fluorescent signals in div10 cortical cultures treated repeatedly with 3 µM Hm in the presence or not of UA and the other test treatments. The lower panel represents Phaco images merged with DHR-123 and TMRM fluorescent signals.

### 3.5. The Anti-Tau Effect of UA Is Not Reproduced Either by Its Immediate Precursor Xanthine or Its Oxidized Metabolite, Allantoin

**Figure 6:**
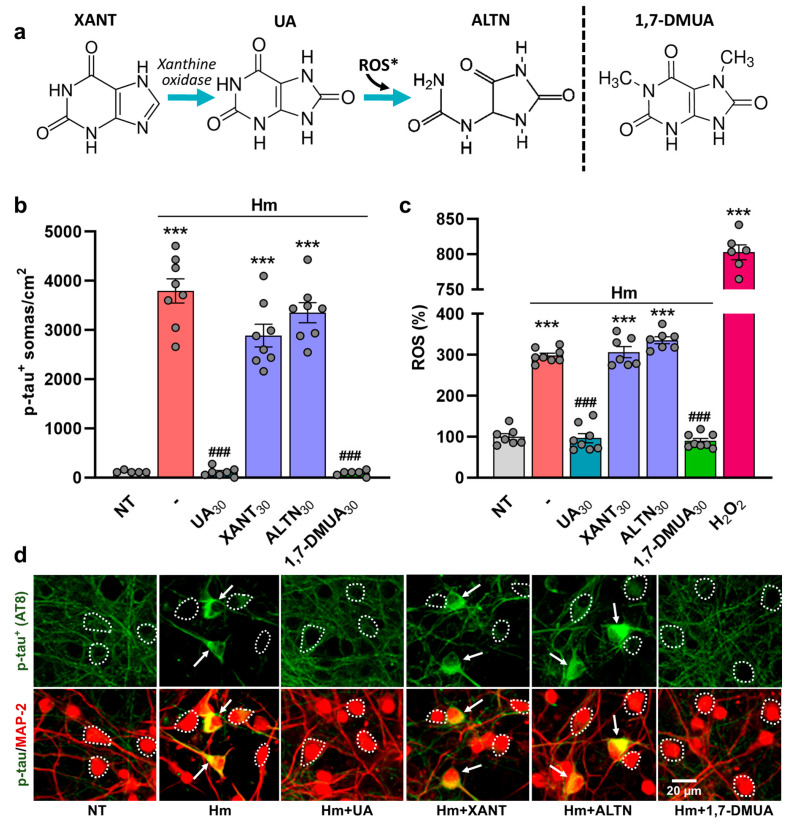
Inhibition of Hm-mediated p-tau accumulation by UA is not reproduced by xanthine or allantoin. (**a**) Simplified metabolic pathway of purine degradation with the chemical structures of UA, XANT, and ALTN. DMUA is a synthetic analog of UA. * In humans, the presence of ALTN reflects the non-enzymatic oxidative catabolism of UA. ROS: reactive oxygen species. (**b**) Estimation of p-tau accumulation (AT8) in div10 cortical cultures treated repeatedly at div7, 8, and 9 with Hm (3 µM) in the presence or not of UA (30 µM), XANT (30 µM), ALTN (30 µM), or DMUA (30 µM). (**c**) ROS emission in div10 cortical cultures undergoing the same treatments as in (**b**). The oxidizer H_2_O_2_ (50 µM) applied acutely for 4 h to the cultures is used as a positive control for ROS emission. (**b**,**c**) Data expressed in numbers of p-tau^+^ somas/cm^2^ (**b**) or ROS levels (**c**) in div10 cultures are presented as the mean ± SEM. One-way ANOVA followed by post hoc Tukey’s test: *** *p* < 0.001 vs. NT, ^###^ *p* < 0.001 vs. Hm. (**d**) Upper panel: Representative microphotographs showing the impact of test treatments on p-tau accumulation in div10 cortical cultures exposed to 3 µM Hm in the presence or not of the different purine derivatives. Lower panel: Same cell culture field where the p-tau immunosignal is combined with MAP-2 immunolabeling. White arrows point to cortical neurons accumulating p-tau in their somas. The white dotted line shows the boundaries of neuronal cell bodies with a near absence of p-tau immunostaining.

We also tested whether the anti-tau and antioxidant effects of UA could be reproduced by its immediate precursor XANT (30 µM) or its oxidation product ALTN (30 µM) (Figure 6a). Neither of these two compounds could reproduce the anti-tau effects of UA (Figure 6b). In contrast, the synthetic 1,7-dimethyl derivative of UA DMUA (30 µM) retained the anti-tau effects of its natural parent compound. We also found that DMUA (30 µM) efficiently curtailed ROS production in Hm (3 µM)-treated cortical cultures. Its efficacy was similar to that of UA (30 µM). In contrast, XANT (30 µM) and ALTN (30 µM) were ineffective (Figure 6c). Figure 6d describes the impact of UA (30 µM), XANT (30 µM), ALTN (30 µM), and DMUA (30 µM) on p-tau accumulation induced by 3 µM Hm in div10 cortical cultures.

### 3.6. The Repressive Action of Uric Acid Against Hm-Induced P-Tau Accumulation Is Mimicked by DOT, a Non-Antibiotic Oxytetracycline Derivative

**Figure 7:**
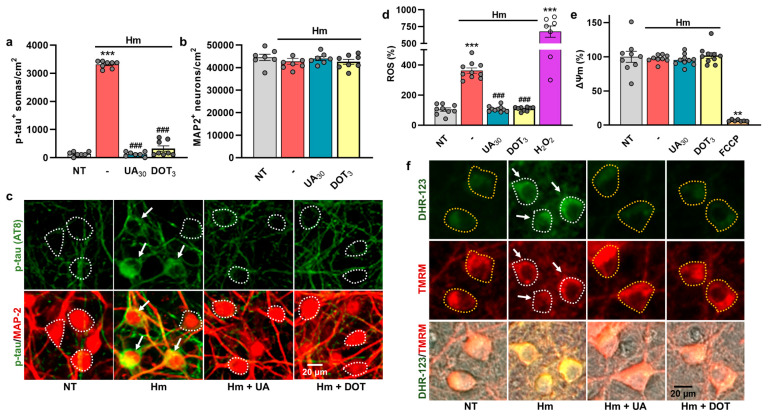
The inhibitory effect of UA against Hm-induced p-tau build-up is mimicked by DOT, a non-antibiotic oxytetracycline derivative. (**a**) Estimation of p-tau accumulation (AT8 immunosignal) in div10 cortical cultures treated repeatedly at div7, 8, and 9 with 3 µM Hm in the presence or not of UA (30 µM) or DOT (3 µM). (**b**) Estimation of neuronal survival in div10 cortical cultures undergoing the same treatments as in (**a**). (**a**,**b**) Data expressed in numbers of p-tau^+^ somas/cm^2^ or neuronal cells/cm^2^ in div10 cultures are presented as the mean ± SEM. One-way ANOVA followed by Tukey’s test. *** *p* < 0.001 vs. NT, ^###^ *p* < 0.001 vs. Hm. (**c**) Upper panel: Representative microphotographs showing the impact of test treatments on p-tau accumulation in div10 cortical neurons in culture exposed repeatedly to 3 µM Hm in the presence or not of the treatments described in (**a**). Lower panel: Same cell culture field where the p-tau immunosignal is combined with MAP-2 immunolabeling. White arrows point to cortical neurons accumulating p-tau in their somas. The white dotted line shows the boundaries of neuronal cell bodies with a near absence of p-tau immunostaining. (**d**) ROS emission in div10 cortical cultures treated repeatedly at div7, 8, and 9 with 3 µM Hm in the presence or not of 3 µM of DOT or 30 µM of UA. Acute exposure to H_2_O_2_ (50 µM) is used as a positive control for ROS emission. Data expressed in % of NT cultures are presented as the mean ± SEM. One-way ANOVA followed by Tukey’s test. *** *p* < 0.001 vs. NT and ^###^ *p* < 0.001 vs. Hm. (**e**) Estimation of ΔΨm in div10 cortical cultures treated repeatedly at div7, 8, and 9 with 3 µM Hm in the presence or not of the treatments described in (**d**). An acute exposure to FCCP (0.5 µM) is used as a reference treatment to promote ΔΨm dissipation. Data expressed in % of NT cultures are presented as the mean ± SEM. Kruskal–Wallis followed by Dunn’s test: ** *p* < 0.01 vs. NT. (**f**) Representative images showing DHR-123 (upper panel) and TMRM (mid panel) fluorescent signals in div10 cortical cultures treated repeatedly with 3 µM Hm in the presence or not of UA (30 µM) or DOT (3 µM). The lower panel represents Phaco images merged with the DHR-123 and TMRM fluorescent signals. Neuronal cell bodies exhibiting increased ROS levels are delineated by a white dotted line and pointed out by a white arrow. Neuronal cell bodies exhibiting basal ROS levels are delineated by a yellow dotted line.

We tested whether a non-antibiotic TC compound that chemically derives from oxytetracycline could mimic the anti-tau effects of UA. This compound is of particular interest in the context of this study, as it demonstrated promising neuroprotective/antioxidant properties in another culture setting that mimics dopamine cell death in PD [28]. Specifically, we used a concentration of DOT of 3 µM reported to provide optimal rescue to dopamine neurons and compared its efficacy to that of UA, at 30 µM. Figure 7a shows that 3 µM of DOT efficiently mimicked the anti-tau effects of 30 µM UA. None of the test treatments had a significant impact on neuronal survival under the present experimental conditions (Figure 7b). The impact of the previous treatments on Hm-induced p-tau accumulation is illustrated by microphotographs in Figure 7c. In line with these observations, we found that DOT (3 µM) was as effective as UA (30 µM) in reducing ROS emission induced by Hm (3 µM) (Figure 7d). As expected, H_2_O_2_ (50 µM) used as a reference prooxidant treatment led to a strong increase in ROS emission. ΔΨm remained unchanged when Hm was applied to the cultures in the presence or not of UA or DOT (Figure 7e). As expected, there was a drop in ΔΨm when FCCP (0.5 µM) was used as a reference treatment to induce mitochondrial membrane depolarization. The results are illustrated by representative images in Figure 7f.

### 3.7. Glutamate-Mediated Tau Neurodegenerative Events Are Similarly Preventable by UA and DOT

**Figure 8:**
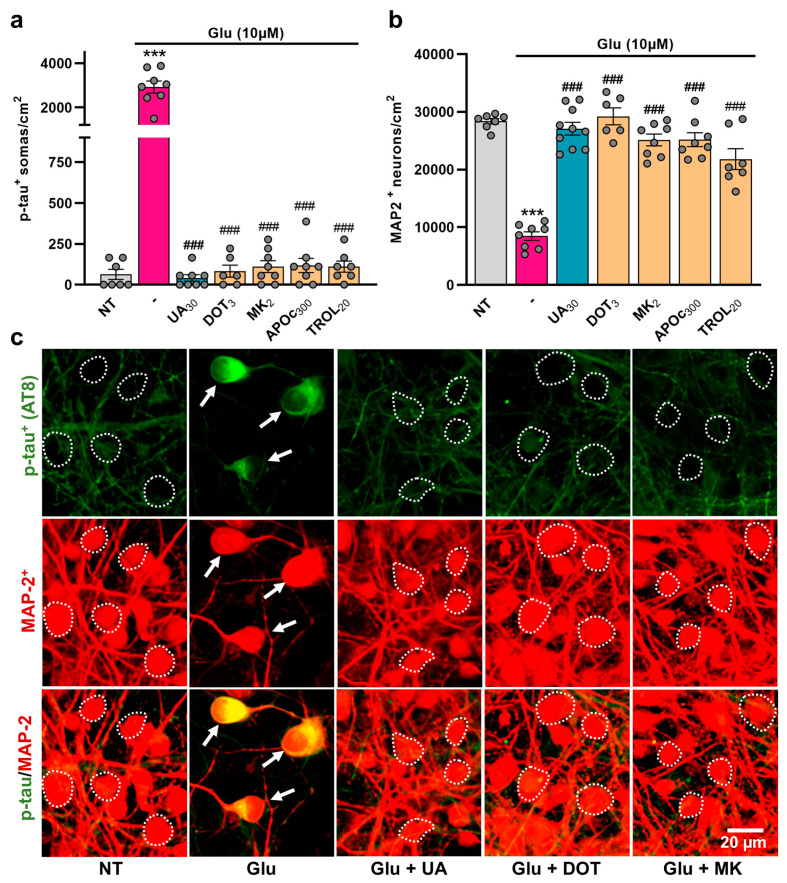
Glutamate-mediated tau neurodegenerative events are preventable by uric acid and the non-antibiotic tetracycline DOT. (**a**) Number of p-tau^+^ cell bodies in div15 cortical cultures that had been previously exposed for 24 h to 10 µM glutamate in the presence or the absence of UA (30 µM), DOT (3 µM), MK-801 (MK, 2 µM), APOc (300 µM), or TROL (20 µM). (**b**) Estimation of neuronal survival in div15 cortical cultures undergoing the same treatments as in (**a**). (**a**,**b**) Data expressed in numbers of p-tau^+^ somas/cm^2^ or neuronal cells/cm^2^ are presented as the mean ± SEM. One-way ANOVA followed by Tukey’s test. *** *p* < 0.001 vs. NT and ^###^ *p* < 0.001 vs. glutamate, only. (**c**) Representative microphotographs showing the impact of test treatments on p-tau accumulation in div15 cortical neuronal cultures challenged previously for 24 h with 10 µM glutamate in the presence or the absence of the treatments described in (**a**). White arrows point to cortical neurons accumulating p-tau in their somas. The white dotted line shows the boundaries of neuronal cell bodies with a near absence of p-tau immunostaining.

We aimed to further investigate whether the inhibitory effects of UA and DOT on p-tau build-up could be observed in another context relevant to neurodegenerative tauopathies. To do this, we tested the impact of these compounds on div14 cortical cultures exposed to an excitotoxic stimulus of moderate intensity [48]. Precisely, we found that a 24 h challenge with 10 µM glutamate caused a robust increase in the number of p-tau^+^ neurons in cortical neurons surviving the excitotoxic insult (Figure 8a). UA (10 µM) totally prevented this increase as well as neuronal loss resulting from excitotoxic stress (Figure 8a,b). The non-antibiotic TC DOT (3 µM) reproduced the anti-tau and neuroprotective effects of UA (Figure 8a,b). The NMDA glutamate receptor blocker, MK-801 (2 µM), the lipid peroxidation inhibitor TROL (20 µM), and the inhibitor of NADPH oxidase APOc (300 µM) also efficiently prevented glutamate-mediated tau neurodegenerative changes.

### 3.8. Potential of UA and DOT to Prevent Tau Amyloid Aggregation

**Figure 9:**
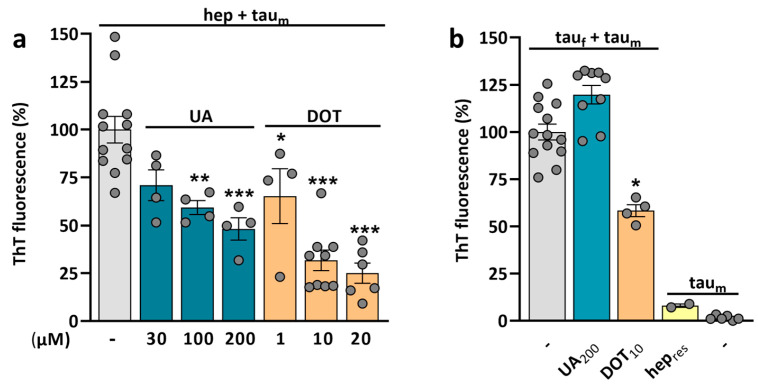
Comparison of the efficacy of uric acid and DOT in limiting tau amyloid aggregation induced by heparin or heparin-assembled tau fibrils. (**a**) Quantitation of heparin-induced tau aggregation in the presence or the absence of UA or DOT. Samples containing 22 µM of monomeric 2N4R tau together with 0.2 mg/mL heparin and 10 µM ThT are incubated for 72 h in the presence or the absence of UA (30, 100, and 200 µM) or DOT (1, 10, and 20 µM) using constant orbital agitation at 600 rpm. Data expressed in % of maximal ThT fluorescence at the endpoint stage are presented as mean values ± SEM. One-way ANOVA followed by post hoc Dunnett’s test. * *p* < 0.05, ** *p* < 0.01, *** *p* < 0.001 vs. hep + tau_m_. (**b**) Tau aggregation induced by 2.2 µM (monomer equivalent) heparin-assembled tau fibrils monitored in the presence or the absence of UA (200 µM) or DOT (10 µM) using the same incubation conditions as before. hep_res_: residual concentration of heparin (0.02 mg/mL) estimated to be present in tau samples when tau fibrils are used as seeds. Data expressed in % of maximal ThT fluorescence at the endpoint stage are presented as mean values ± SEM. Kruskal–Wallis with a post hoc Dunn’s test. * *p* < 0.05 vs. tau_f_ + tau_m_.

The efficacy of UA and DOT in limiting p-tau build-up led us to determine whether these two molecules could also operate as inhibitors of tau aggregation (Figure 9). For that, we established a ThT fluorescence assay in which the aggregation of 2N4R tau_m_ is seeded by heparin (0.2 mg/mL), a polyanion commonly used as a cofactor to seed tau aggregation [24,30,49]. The estimation of steady-state ThT fluorescence levels after 72 h of continuous orbital agitation of tau samples revealed that UA and DOT significantly reduced tau aggregation (Figure 9a). The inhibitory effect of UA was significant at 100 µM and optimal at 200 µM. For DOT, we noted a substantial reduction in tau aggregation at 1 µM, with optimal inhibitory effects at 20 µM. To complete this characterization, we investigated the capacity of UA and DOT to reduce tau aggregation induced by 2.2 µM (monomer equivalent) heparin-assembled tau fibrils (tau_f_), i.e., experimental conditions where tau aggregation does not directly rely on heparin as a cofactor (Figure 9b). In this context, 10 µM DOT was able to significantly reduce tau aggregation, while UA did not show inhibitory effects at 200 µM. Note that tau aggregation was minimal when tau_m_ were mixed with residual levels of heparin (0.02 mg/mL) present in test samples containing heparin–tau fibrils. As expected, when tau_m_ were agitated in the absence of heparin or tau_f_, fibrillation did not occur.

## 4. Discussion

To replicate early tau neuropathological changes occurring in AD and other tauopathies, we utilized mouse primary cortical cultures, which were exposed to redox-active iron from Hm, a breakdown product of Hb. We show that Hm significantly promoted neuronal accumulation of pathological hyperphosphorylated tau. We established that UA, the end-product of human purine metabolism, effectively prevented this process by suppressing iron-mediated ROS emission. In contrast, UA’s immediate precursor, XANT, and its oxidation product, ALTN, were ineffective. The anti-tau and antioxidant effects of UA were successfully reproduced by DOT, a non-antibiotic TC derivative of oxytetracycline that is structurally unrelated to purine compounds. Both UA and DOT were also effective when p-tau build-up was induced by moderate excitotoxic stimulation with glutamate. Using a ThT aggregation assay to mimic tau aggregation at more advanced stages of tauopathies, we demonstrate that UA and DOT could limit tau aggregation seeded by heparin. However, only DOT was effective when the aggregation process was induced by heparin-assembled tau fibrils.

### 4.1. Hemin Promotes Pathological P-Tau Accumulation in Cultured Cortical Neurons

Our initial objective was to model early cellular tau abnormalities that may occur before the formation of tau NFTs [1,50,51]. To achieve this, we established a culture system of mouse cortical neurons in which pathological p-tau accumulation is induced by redox-active iron from Hm, a degradation product of Hb. We chose this approach because several studies suggest that iron dyshomeostasis might actively contribute to the progression of tau lesions in AD pathology and other tauopathies [52,53,54], including chronic traumatic encephalopathy, a type of tauopathy arising from repeated traumatic head injuries and blood leakage around small vessels [8,55]. Brain imaging analyses provide the most compelling evidence for this hypothesis, showing that iron deposition is correlated with tau aggregates and neuronal loss in the brains of individuals diagnosed with AD [20]. A study by Yamamoto and colleagues further supports this hypothesis by indicating that iron (III) binds to hyperphosphorylated tau aggregates in tissue extracts from AD-affected brains [21].

Specifically, we monitored p-tau accumulation using the AT8 antibody, which detects a key phosphorylated epitope (Ser202/Thr205) associated with AD and other tauopathies [16,24]. The relevance of the AT8 antibody lies in its use for tracking the progression of the tau pathology both before and after the formation of tau NFTs [1,50,51].

We established that treatment with Hm over three consecutive days resulted in a concentration-dependent increase in the AT8 immunosignal within a significant portion of cortical neurons in culture. The accumulation of p-tau was generally detectable in both the soma and neuritic extensions, aligning with neuropathological findings in AD and other tauopathies [56,57]. The present observations are also consistent with data reported by Wan and colleagues [58], who demonstrated that hyperphosphorylated tau accumulates in cultured cortical neurons maintained in a medium enriched with soluble iron. Interestingly, the same authors found that mice on a high-iron diet exhibited pathological tau accumulation in the cortex in conjunction with cognitive deficits. The fact that only a fraction of cortical cell bodies accumulated p-tau upon treatment with Hm is consistent with neuropathological reports indicating that tau lesions affect only specific subsets of vulnerable neurons in tauopathies [59,60,61]. Interestingly, we found that Hm was capable of promoting p-tau accumulation in neuronal cell bodies without causing concurrent neuronal loss under specific conditions of treatment. Precisely, at a concentration of 3 µM of Hm, which did not lead to neuronal death, the accumulation of p-tau was observable in about 8–10% of neuronal somas. Since early tau neuropathological changes can cause neuronal dysfunction without leading to significant neurodegeneration [60,62,63], we decided to implement a treatment regimen with 3 µM of Hm to study the effects of tau-targeted therapies.

### 4.2. UA, the End Metabolic Product of Purines in Humans, Prevents Hm-Induced P-Tau Build-Up

Elevated circulating levels of UA—the final breakdown product of purine nucleotides in humans—play a significant role in gout and kidney stone formation. A high UA level in the blood is also often regarded as a potential cardiovascular risk factor [64]. However, several studies have reported that a reduction in serum UA is associated with an increased risk of AD dementia and other tauopathies [26,27,65]. This prompted us to investigate whether UA could influence the accumulation of pathological p-tau in our treatment paradigm with Hm. Our results demonstrate that UA very efficiently inhibited Hm-induced p-tau accumulation across a wide range of concentrations (0.1–300 µM), suggesting that restoring UA serum levels to baseline may be of therapeutic value for tauopathies. To further investigate the nature of UA’s anti-tau effects, we used a working concentration of 30 µM, which reflects the levels of UA typically found in human cerebrospinal fluid, while plasma levels are generally more than 10-times higher [66,67].

### 4.3. UA Exerts Anti-Tau Effects by Curtailing Hm-Mediated Oxidative Stress

UA constitutes approximately 30% to 50% of the body’s normal antioxidant capacity, making it a crucial physiological antioxidant [45,46,67,68]. This prompted us to investigate whether UA’s suppressive effects on p-tau accumulation were due to its antioxidant properties. The use of the fluorogenic probe DHR-123 revealed that ROS levels increased in neurons treated with Hm and that UA prevented this effect, confirming the view that oxidative stress was responsible for p-tau accumulation in this setting. This conclusion aligns with data from other studies [58,69,70], although ROS have also been reported to promote tau dephosphorylation under certain conditions [71].

The essential role of ROS in pathological p-tau accumulation in our model system was further supported by the fact that an acute challenge with H_2_O_2_ led to the accumulation of the AT8 immunosignal in a subpopulation of cortical neurons. Unlike what we observed with Hm, UA was unable to prevent p-tau build-up or ROS production triggered by H_2_O_2_, despite its known ability to scavenge this oxidizing agent in in vitro studies [72]. One might assume that in neuron-enriched cultures, the antioxidant properties of UA are likely to be overwhelmed by the severity and acute nature of the insult elicited by H_2_O_2_. Our finding aligns with a previous report indicating that differentiated NSC-34 motor neuron-like cells exposed to H_2_O_2_ are not protected from degeneration when receiving urate as concomitant treatment [73]. Indeed, only a conditioned medium from UA-treated astrocytes was found to be protective against damage caused by H_2_O_2_ [73].

The concurrent use of the mitoprobe TMRM with the ROS indicator DHR-123 revealed that ΔΨm remained unaffected by Hm treatment, whether UA was present or not in the cultures. This suggests that the generation of ROS and the accumulation of p-tau induced by Hm were not directly linked to mitochondrial dysfunction. This stands in apparent contradiction to previous reports suggesting that a p-tau build-up could result from mitochondrial disturbances [74,75,76]. However, this may simply suggest that ROS from mitochondrial and non-mitochondrial origins may equally generate tau lesions.

### 4.4. UA Exerts Anti-Tau Effects by Preventing Hm-Mediated Lipid Peroxidation

Interestingly, DESF, which has iron-chelating properties [77], along with TROL and LIP—both of which inhibit lipid peroxidation and ferroptosis [78]—were all able to replicate the anti-tau and ROS inhibitory effects of UA. This suggests that UA exerts its anti-tau effects by disrupting a sequence of events in which the reaction between Fe(II) and H_2_O_2_—the Fenton reaction—produces hydroxyl radicals, subsequently leading to lipid peroxidation [79,80]. In line with this hypothesis, Hm has been shown to act as a catalyst for lipid peroxidation [81,82], whereas UA was reported to operate as an inhibitor of this process [72,83,84], possibly by scavenging hydroxyl radicals [45,85]. Additionally, the antioxidant capacity of UA may depend on its ability to reduce redox iron levels by forming coordination complexes with iron ions [86]. Due to limited access of Hm to cells [87], UA may primarily exert its ROS suppressive effect at the outer leaflet of the plasma membrane. This action may prevent ROS propagation into the intracellular compartment, thereby inhibiting p-tau accumulation. Note that this proposed scenario does not totally exclude that UA could also operate by stimulating neuronal iron efflux through the amyloid protein precursor/ferroportin complex as described before in another experimental setting [88].

It is important to note that the glycoprotein APO, which has a high capacity for iron chelation, and VitC, known for its strong antioxidant properties, failed to prevent p-tau accumulation in this context. The lack of inhibitory effects of vitamin C on Hm-induced tau accumulation may be linked to the fact that oxidative stress originates in the lipid environment of the plasma membrane. Indeed, VitC is known to be more effective as an antioxidant in aqueous environments than in lipid environments [89]. The lack of anti-tau effects observed with APO is somehow surprising, as we established previously that this glycoprotein is highly effective in preventing iron-mediated lipid peroxidation in other experimental settings [28,35,46]. We can assume that while ferric iron in the heme pocket of Hm remains accessible to low-molecular-weight molecules like DESF, it is not accessible, however, to large proteins with high molecular mass, such as APO [90].

### 4.5. The Anti-Tau Effect of UA Is Not Reproduced by Either Its Immediate Precursor XANT or Its Oxidative Product ALTN

To identify the structural features of UA that contribute to its antioxidant/anti-tau properties, we compared its effects with those of its immediate precursor XANT, which is produced through the degradation pathway of adenosine or guanosine [91]. Consistent with findings reported by Muraoka and Miura [83], our results indicate that XANT lacks intrinsic antioxidant properties. This likely explains why, in contrast to UA, XANT does not exert anti-tau effects in our model system. UA is chemically defined as 2,6,8-trioxy-purine and differs from XANT only by the presence of an 8-oxo group on the imidazole ring of the purine structure [92]. This suggests that this functional group contributes to UA’s antioxidant and anti-tau activities. Supporting this observation, the 1,7-dimethyl derivative analogue of UA, which retains an 8-oxo group on its purine structure, was equally as effective as UA in preventing tau accumulation and oxidative stress caused by Hm exposure. Note that the oxidative product of UA ALTN—a pyrimidine ring-opened derivative—did not reduce p-tau burden, indicating that only an intact 2,6,8-trioxy-purine structure can provide anti-tau and antioxidant effects.

### 4.6. DOT, a Non-Antibiotic Oxytetracycline Derivative, Mimics the Anti-Tau Effects of UA

Compounds that can mimic the antioxidant effects of UA and penetrate the brain may offer a promising therapeutic option for reducing the pathological p-tau load. Non-antibiotic TC compounds, which have demonstrated neuroprotective and antioxidant properties in other studies, meet these criteria [28,35,93,94]. Here, we aimed to compare the anti-tau activity of UA with that of DOT, a non-antibiotic TC derived from oxytetracycline, which demonstrated potent antioxidant and neuroprotective properties in a culture system that models dopamine cell death in PD [28]. We found that DOT prevented p-tau accumulation induced by Hm with a similar efficacy as UA. As expected, the anti-tau effects of DOT were correlated with its ability to counteract oxidative stress induced by Hm.

### 4.7. P-Tau Build-Up Induced by Glutamate Is Preventable by UA and DOT

We wanted to investigate whether the suppressive action of UA and DOT against Hm-induced p-tau build-up could be observed in another experimental setting similarly relevant for neurodegenerative tauopathies. In addition to oxidative stress, neuronal hyperexcitability is suspected to contribute to tau pathological events in AD and other tauopathies [22,23,95]. This led us to implement a paradigm in which pathological p-tau build-up is triggered by an excitotoxic stimulation with the neurotransmitter glutamate [48,96].

After a 24 h challenge with 10 µM glutamate, we found that the AT8 immunosignal was robustly increased in the soma of a subpopulation of cortical neurons that survived the excitotoxic insult. Most interestingly, UA and DOT effectively suppressed p-tau build-up and neurodegenerative events associated with glutamate exposure. These results are consistent with earlier studies showing that UA [97,98] and certain TCs [35,99] can modulate glutamate-mediated neurotoxicity.

The NMDA receptor blocker MK-801, the inhibitor of the superoxide-generating enzyme NADPH oxidase APOc, and the inhibitor of lipid peroxidation TROL also prevented tau abnormalities caused by glutamate. This indicates that UA and DOT exerted their anti-tau effects by primarily blocking a mechanism conveyed by NMDA receptors, and secondarily by lipid peroxidation by-products induced through NADPH oxidase activation. These observations align with previous research studies demonstrating that NADPH oxidase is a major source of NMDA-induced superoxide formation in neurons [100,101,102]. This also indirectly confirms that ROS can cause tau lesions, regardless of the mechanisms that produce them. Whether UA and DOT modulated NMDA receptor activity or limited intracellular ROS production in response to receptor activation remains to be determined. Note that the inhibition of glutamate-mediated p-tau build-up by UA, DOT, and the other test compounds was closely linked to their capacity to prevent neuronal loss. This differs from what we observed in the Hm paradigm, where p-tau accumulation was preventable under conditions that do not result in neuronal death.

### 4.8. UA and DOT Have the Potential to Restrain Tau Aggregation

Following exposure to Hm or glutamate, we could not visually detect the presence of aggregates in cell bodies accumulating p-tau. This indicates that while the mouse tau protein can form amyloid fibrillar aggregates similar to the human tau protein [103], the post-translational modifications occurring in AT8^+^ mouse tau species are not sufficient to promote aggregation under the present culture conditions.

Therefore, to investigate whether UA and DOT could also act as inhibitors of tau amyloid aggregation, we implemented a ThT-based aggregation assay using human recombinant 2N4R tau_m_ with the polyanion heparin operating as a cofactor for aggregation [30,104]. While this is an artificial model system designed to mimic tau aggregation in vitro, it may be valuable for identifying tau anti-aggregant molecules [30].

We found that both UA and DOT effectively reduced heparin-induced tau aggregation. However, DOT was significantly more potent than UA, with the lowest effective concentrations being estimated at 1 µM for DOT and 100 µM for UA. In addition, we investigated whether UA and DOT could reduce the tau aggregation induced by heparin-induced tau fibrils, i.e., conditions where heparin does not directly operate as a cofactor for tau fibrillation. While DOT remained effective in this context, UA did not. This suggests that DOT and UA reduced tau amyloidogenesis through distinct mechanisms.

The absence of anti-aggregant effects of UA when tau fibrillation was seeded with tau fibrils instead of heparin suggests that UA may reduce tau fibrillation by direct interaction with the cofactor heparin. This is notably supported by past studies showing the adsorption of heparin on sodium urate [105]. It has been proposed that the TC antibiotic doxycycline, which is closely related to DOT, could prevent tau aggregation by interacting with the 4R-repeat domain of the protein [29]. Therefore, we can assume that DOT could operate similarly; however, further research is necessary to confirm this point.

## 5. Conclusions

In conclusion, we have established a model system of neuronal cortical cultures in which low-intensity stressors serve to simulate pathological tau phosphorylation in tauopathies through a mechanism involving oxidative stress. Our findings show that UA, the end-product of purine catabolism in humans, and DOT, a non-antibiotic derivative of oxytetracycline chemically unrelated to purine compounds, can efficiently suppress p-tau build-up through their antioxidant potential. Additionally, we found that DOT and, to a lesser extent, UA, could reduce tau amyloid aggregation in a ThT aggregation assay. Overall, our results indicate that treatments involving urate-elevating agents might be therapeutically beneficial in preventing tau abnormalities associated with tauopathies. The non-purine TC compound DOT may represent an interesting alternative to UA-related therapies.

## Data Availability

Data is contained within the article. Further inquiries can be directed to the corresponding author.

## Abbreviations

AD	Alzheimer’s disease
ACM	astrocyte-conditioned medium
ALTN	allantoin
cNb	complete neurobasal medium
ΔΨm	mitochondrial membrane potential
DESF	desferioxamine
DHR-123	dihydrorhodamine-123
DMEM	Dulbecco’s Modified Eagle’s Medium
div	day(s) in vitro
DMUA	1,7-dimethyluric acid
DOT	12a-deoxy-dedimethylamino-oxytetracycline
FCCP	carbonyl cyanide 4-(trifluoromethoxy) phenylhydrazone
Hb	hemoglobin
Hm	hemin
LIP	Liproxstatin-1
NFTs	neurofibrillary tangles
PBS	Dulbecco’s phosphate-buffered saline
Phaco	phase contrast
ROS	reactive oxygen species
tau_f_	tau fibrils
tau_m_	tau monomers
TC	tetracycline
TMRM	tetramethylrhodamine methyl ester
TROL	trolox
UA	uric acid
XANT	xanthine
